# Physical Activity During Recess Outdoors and Indoors Among Urban Public School Students, St. Louis, Missouri, 2010–2011

**DOI:** 10.5888/pcd10.130135

**Published:** 2013-11-21

**Authors:** Irene Tran, B. Ruth Clark, Susan B. Racette

**Affiliations:** Author Affiliations: Irene Tran, B. Ruth Clark, Washington University School of Medicine, St. Louis, Missouri.

## Abstract

We measured the quantity and intensity of physical activity in 106 urban public school students during recess outdoors, recess indoors in the gym, and recess indoors in the classroom. Students in grades 2 through 5 wore accelerometer pedometers for an average of 6.2 (standard deviation [SD], 1.4) recess periods over 8 weeks; a subsample of 26 also wore heart rate monitors. We determined, on the basis of 655 recess observations, that outdoor recess enabled more total steps per recess period (*P* < .0001), more steps in moderate-to-vigorous physical activity (*P* < .0001), and higher heart rates than recess in the gym or classroom. To maximize physical activity quantity and intensity, school policies should promote outdoor recess.

## Objective

Low physical activity levels contribute to poor physical fitness and are associated with cardiovascular disease risk factors in children (1,2). The *2008 Physical Activity Guidelines for Americans* recommends that children engage in at least 60 minutes of physical activity daily, most of which should be moderate-to-vigorous physical activity (MVPA) (3). School-based physical activity has a positive effect on students’ health and academic outcomes, particularly among low-income and minority children (4). The objective of this study was to compare the quantity and intensity of physical activity achieved during outdoor and indoor recess among urban elementary schoolchildren.

## Methods

This observational study was conducted from December 2010 through March 2011. Students in grades 2 through 5 attending 2 urban public schools in St. Louis were invited to participate during classroom presentations about the study. Of the 233 invited students, 113 provided oral consent and written parental consent and were enrolled.

Primary outcomes were total steps and steps rated MVPA during recess in 3 locations (outdoors, gym, and classroom) determined using Omron HJ-151 uniaxial accelerometer pedometers (Omron Healthcare, Inc, Lake Forest, Illinois). MVPA was defined as step cadence of 3 miles per hour or more, based on a proprietary Omron formula. Students wore the pedometers at the hip throughout each 20-minute recess period, 2 to 3 times weekly during 3 weeks over an 8-week period. Recess choices included free play (eg, jump rope, Frisbee, ball games) and structured activities (eg, hip-hop dance, kickball, classroom games, reading), with location dictated by weather conditions and school policy. Research personnel recorded step counts at the recess location after each recess period.

Heart rate during recess was assessed as an estimate of physical activity intensity using Polar E600 heart rate monitors (Polar Electro, Lake Success, New York) in a random sample of 26 students. Heart rate was recorded every 5 seconds and physiologic values (ie, 50–215 beats per minute [BPM]) were averaged.

Health assessments were conducted on 2 occasions and included resting heart rate and blood pressure measured with Omron professional automated monitors (Omron Healthcare, Inc, Lake Forest, Illinois), weight on a digital scale (Health-o-Meter, Bridgeview, Illinois), height with a stadiometer (Seca, Birmingham, United Kingdom), and waist circumference at the superior border of the iliac crest. HealthWatch Pro 3.1 software (Seattle, Washington) was used to compute sex- and age-specific percentiles for body mass index (BMI) (5) and waist circumference, and sex-, age-, and height-specific percentiles for blood pressure (6). This study was approved by the Washington University School of Medicine Human Research Protection Office and the school district’s Research Review Committee.

Generalized estimating equations with an exchangeable correlation matrix were used to evaluate the effects of recess location and sex on total steps and steps in rated MVPA (SAS version 9.3 [SAS Institute, Inc, Cary, North Carolina]). Analysis of variance was used to compare heart rate between recess locations.

## Results

Of 113 students enrolled in this study, 106 students provided pedometer and health assessment data required for inclusion in the analyses. Participants were 53.8% female, 88.7% black, and aged from 7.5 years to 12.3 years (mean, 9.8 y, standard deviation [SD], 1.2), with a mean resting heart rate of 88 BPM (SD, 11; range 55–121). Regarding risk prevalence, 9.8% of participants were classified prehypertensive or hypertensive (blood pressure ≥90th percentile), 11.4% were overweight (BMI ≥85th and <95th percentile), 28.6% were obese (BMI ≥95th percentile), and 18.4% were abdominally obese (waist circumference ≥90^th^ percentile).

**Figure 1:**
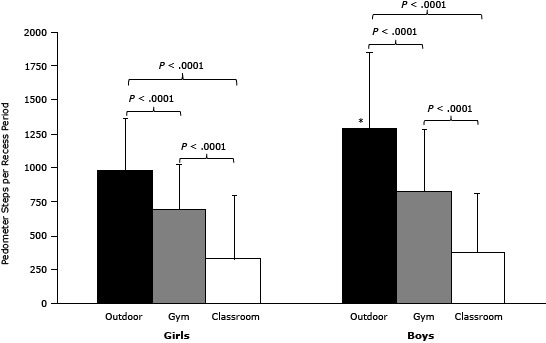
Pedometer-recorded steps by recess location among school students in St. Louis, Missouri, 2010–2011. Bars represent steps per recess period by recess location (mean, standard deviation) averaged across 106 students. The number of recess observations was 291 outdoors, 196 in the gym, and 168 in the classroom. *Boys took more steps during outdoor recess than did girls (*P* < .0001). **Table:** 

On the basis of 655 recess observations of 106 students (6.2 recess periods per student; SD, 1.4), we determined that the greatest quantity and intensity of physical activity were achieved during outdoor recess. There was a significant interaction between recess location and sex on steps per recess period (*P* = .007). Girls took an average of 976 steps per recess period outdoors (SD, 385), 692 steps in the gym (SD, 330), and 328 steps in the classroom (SD, 465) (*P* < .0001 for all comparisons) (Figure 1). Boys took 1,281 steps per recess period outdoors (SD, 566), 824 steps in the gym (SD 459), and 378 steps in the classroom (SD, 433, *P* < .001 for all comparisons). Boys accumulated more steps than girls during recess outdoors (*P* < .001).

Sex	Outdoor, Mean (SD)	Gym, Mean (SD)	Classroom, Mean (SD)
**Girls**	976 (385)	692 (330)	328 (465)
**Boys**	1,281 (566)	824 (459)	378 (433)

More steps during outdoor recess were rated MVPA (56%) than steps during recess in the gym (36%) or classroom (15%) (*P* <.001 across recess locations).

**Figure 2:**
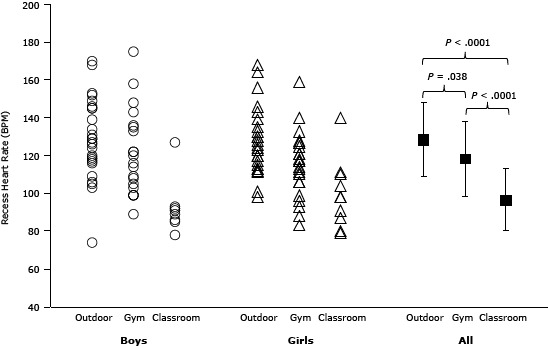
Heart rate by recess location among school students in St. Louis, Missouri, 2010-2011. Each circle and triangle represents a single recess observation (ie, an individual student’s average heart rate during a single recess period). Black squares represent the mean and standard deviation heart rate of the 26 students in each location. **Table:** 

We acquired individual and mean heart rate data for 111 recess observations (4.3 recess periods per student [SD, 2.4] among 26 students) (Figure 2). Average heart rate differed by recess location *(P* < .0001): 128 BPM (SD, 20) outdoors, 118 BPM (SD, 20) in the gym, 97 BPM (SD, 17), in the classroom.

Sex	Outdoor, Mean (SD)	Gym, Mean (SD)	Classroom, Mean (SD)
**Girls**	129 (21)	122 (22)	93 (15)
**Boys**	128 (18)	115 (17)	100 (18)
**All**	128 (20)	118 (20)	97 (17)

## Discussion

Our study revealed that location is a major determinant of the quantity and intensity of physical activity achieved during recess. Students accumulated more total steps and more steps rated MVPA during recess outdoors than during recess in a gym or classroom. Consistent with findings in other studies (7), boys were more active than girls.

High levels of MVPA by children are associated with reduced cardio-metabolic risk factors (8). The Institute of Medicine recommends that schools provide opportunities for students in grades kindergarten through 12 to participate in 60 minutes of physical activity each school day (9). A national sample of children aged 6 to 11 years found that only 42% achieved the recommended amount of daily physical activity (10). Children who live in low-income neighborhoods may have limited opportunities outside school to engage in physical activity. Reznik et al found that students are more active on school days with a physical education class than on those without and on days with outdoor recess than on those without (11). With physical education curricula limited in many public school districts, recess may account for a large portion of school-based physical activity.

Recess provides students with opportunities for important physical, cognitive, social, and emotional benefits (12). Although our results highlight the importance of outdoor recess to optimize physical activity, indoor recess is often necessary and therefore should include creative activities that promote movement of moderate-to-vigorous intensity, whether in a gym or a classroom (13).

Study limitations are inclusion of only 2 schools and the possibility that study participants were more or less active than nonparticipating students, which would limit the generalizability of our results. Furthermore, accelerometer pedometers provided only estimates of students’ MVPA.

In summary, outdoor recess provided the optimal environment for children to engage in quality physical activity. When outdoor recess is not feasible, district and school policies should mandate indoor recess opportunities that promote active play, particularly in urban settings.
